# Baseline gene expression in BALB/c and C57BL/6 peritoneal macrophages influences but does not dictate their functional phenotypes

**DOI:** 10.3389/ebm.2024.10377

**Published:** 2025-01-03

**Authors:** Carlos M. Restrepo, Alejandro Llanes, Lizzi Herrera, Esteban Ellis, Iliana Quintero, Patricia L. Fernández

**Affiliations:** ^1^ Centro de Biología Celular y Molecular de Enfermedades, Instituto de Investigaciones Científicas y Servicios de Alta Tecnología (INDICASAT AIP), Panama City, Panama; ^2^ Sistema Nacional de Investigación (SNI), Secretaría Nacional de Ciencia Tecnología e Innovación (SENACYT), Panama City, Panama; ^3^ Bioterio, Instituto de Investigaciones Científicas y Servicios de Alta Tecnología (INDICASAT AIP), Panama City, Panama; ^4^ Departamento de Biotecnología, Facultad de Ciencias de la Salud, Universidad Latina de Panamá, Panama City, Panama; ^5^ Facultad de Ciencia y Tecnología, Universidad Tecnológica de Panamá, Panama City, Panama

**Keywords:** macrophages, RNA-seq, BALB/c, C57BL/6, pathogen

## Abstract

Macrophages are effector cells of the immune system and essential modulators of immune responses. Different functional phenotypes of macrophages with specific roles in the response to stimuli have been described. The C57BL/6 and BALB/c mouse strains tend to selectively display distinct macrophage activation states in response to pathogens, namely, the M1 and M2 phenotypes, respectively. Herein we used RNA-Seq and differential expression analysis to characterize the baseline gene expression pattern of unstimulated resident peritoneal macrophages from C57BL/6 and BALB/c mice. Our aim is to determine if there is a possible predisposition of these mouse strains to any activation phenotype and how this may affect the interpretation of results in studies concerning their interaction with pathogens. We found differences in basal gene expression patterns of BALB/c and C57BL/6 mice, which were further confirmed using RT-PCR for a subset of relevant genes. Despite these differences, our data suggest that baseline gene expression patterns of both mouse strains do not appear to determine by itself a specific macrophage phenotype.

## Impact statement

In this study, we used RNA-Seq to analyze the baseline gene expression profiles of peritoneal macrophages from C57BL/6 and BALB/c mice, which are known for their Th1- and Th2-biased immune responses, respectively. Our goal was to understand how these baseline patterns influence the interpretation of gene expression changes in other studies where pathogen interaction is considered. We found that, while there are significant differences in the basal gene expression profiles between BALB/c and C57BL/6 peritoneal macrophages, these differences do not dictate the macrophage activation phenotype. Therefore, baseline gene expression in resident peritoneal macrophages should not influence the interpretation of transcriptomic analyses conducted in response to pathogen infection. This finding is crucial for analysing the results of studies comparing gene expression patterns in resident peritoneal macrophages during pathogen challenges. This research underscores important considerations for studying the mechanisms that shape resident peritoneal macrophage phenotypes in response to intracellular pathogens.

## Introduction

Macrophages are cells from the innate immune system that are essential in the elimination of pathogens and the initiation of the immune response. Tissue resident macrophages adapt to their microenvironment and exhibit specific functions essential to maintain tissue homeostasis. Distinct transcriptional regulators determine tissue-specific transcription programs in resident macrophages [1]. Macrophages are usually in a naïve phenotype, which is altered depending on environmental signals. Two main activated states of macrophages have been proposed, the classically activated subtype (M1), which shows a pro-inflammatory and microbicidal profile, and the alternative activated subtype (M2), which is associated with an anti-inflammatory response and tissue repair and homeostasis [2]. Various M2 subsets have been described, induced by the exposure of naïve macrophages to different cytokines, all of which have immunosuppressive features [3]. Polarization into either of these activated states results in the alteration of cell surface marker expression and inflammatory-related factors. The M1 macrophages are characterized by overexpression of the surface markers CD80, CD86 and CD16/32, and actively produce pro-inflammatory cytokines such as the tumour necrosis factor alpha (TNF-α), interleukin 6 (IL-6) and IL-12 [4]. Meanwhile, M2 macrophages have been shown to express higher levels of mannose receptor (CD206), arginase-1, and the anti-inflammatory cytokine IL-10 [4]. Currently, new knowledge in areas such as proteomics and transcriptomics has revealed a more complex scenario within this classification that requires further studies.

Activated lymphocytes can modulate the differentiation states of macrophages through the profile of cytokines that they secret, thus controlling the type of immune response to specific stimuli. For example, Th1 cytokines like TNF-α and interferon gamma (IFN-γ) promote the development of the M1 phenotype, while promotion of the M2 phenotype is directed by Th2 cytokines, usually IL-4 and IL-13 [5]. However, other factors that are not characterized in the context of Th1 Th2 responses elicit similar macrophage phenotypes [6]. Although there are discrepancies between *in vivo* and *in vitro* studies regarding the expression of markers that characterize these phenotypes [7], *in vitro* studies have shed light on the mechanisms involved in the differential response of macrophages to several stimuli.

Laboratory mouse strains C57BL/6 and BALB/c respectively display Th1-biased and Th2-biased immune responses, thus making them interesting models for studying the mechanisms involved in the heterogeneity of macrophage activation. For instance, macrophages isolated from C57BL/6 tend to show higher levels of nitric oxide (NO) production in comparison to BALB/c-derived macrophages after stimulation with IFN-γ and LPS [2, 8, 9]. These innate differences between the two mouse strains have been also reflected in the higher resistance of C57BL/6 against infections to different pathogens such as *Mycobacterium tuberculosis*, *Pasteurella pneumotropica*, *Chlamydia* and *Leishmania* spp. [10–13]. Furthermore, studies on murine macrophage infection with *Leishmania* species have shown that macrophages from Th1-biased strains are more easily activated than those from Th2-biased strains [14, 15]. However, it has also been shown that infection with *Leishmania* can increase the ability of macrophages to stimulate a Th2 response [16]. In fact, a quite heterogeneous range of responses and patterns of gene expression have been reported when studying the immune response of C57BL/6 and BALB/c to different *Leishmania* species [17–22].

During the last few years, our group has been working in characterizing the immune response of BALB/c and C57BL/6 mice to the infection with *Leishmania panamensis* [23, 24]. Our results have shown that BALB/c-infected mice are more susceptible to the infection than C57BL/6, exhibiting higher inflammation and parasite loads at the inoculation site [24]. In a study conducted *in vitro*, we observed that infection of C57BL/6 macrophages with *L. panamensis* induced a pro-inflammatory gene expression pattern associated with a classic M1 phenotype, whereas BALB/c macrophages showed gene expression patterns intermediate between M1 and M2 [23]. In this study, we compared the baseline gene expression patterns from uninfected BALB/c and C57BL/6 macrophages, used as controls in the previous study. Our main goal is to assess if there is a possible predisposition of these mouse strains to the M1 or M2 phenotype, which may in turn affect the interpretation of changes in gene expression patterns associated with the interaction with pathogens such as *Leishmania* spp.

## Materials and methods

### Institutional ethics committee statement and laboratory animal handling

All experimental procedures involving mice were approved by the Institutional Animal Care and Use Committee of INDICASAT (protocol number IACUC-14-002, approved on 30 May 2014, and renewed on 29 July 2019). All procedures were performed following international and institutional regulations for the ethical handling of laboratory animals. Animals were maintained in Specific Pathogen Free (SPF) conditions at INDICASAT’s animal facility with free access to food and water, a constant temperature of 24°C and a 12-hour light/dark cycle.

### Murine macrophage isolation

Resident peritoneal macrophages were obtained from female BALB/c and C57BL/6 mice, by peritoneal washing with chilled Roswell Park Memorial Institute (RPMI) (Gibco, Gaithersburg, MD, United States). A density of 1 × 10^6^ cells per well in 24-well plates were seeded in RPMI with 10% fetal bovine serum, penicillin/streptomycin (100 U/mL/100 μg/mL). Cells were incubated for 2 h at 37°C and 5% CO_2_. After removing non-adherent cells by washing with RPMI medium, adherent macrophages were incubated for 24 h more at 37°C and 5% CO_2_. Peritoneal macrophages were processed independently for each mouse strain and each experiment was performed in triplicate.

### Flow cytometry

After 24 h of culture, cells were harvested, washed with phosphate-buffered saline (PBS), and blocked with 1% BSA for 15 min. After washing, cells were incubated with 5 μg/mL of anti-mouse CD11b FITC, CD80 APC, CD86 PE-Cy5, MRC1 (CD206) APC, MHC-II PE (eBioscience, San Diego, CA, United States) and/or anti-mouse F4/80 PE (Abcam, Cambridge, United Kingdom) diluted in 1% BSA, for 30 min at 4°C. Following two sequential washes, cells were resuspended in PBS for flow cytometry analysis. Events were acquired with a CyFlow cytometer, and the data were analyzed using FloMax software (PARTEC, Münster, Germany) and FlowJo v10.10 (Becton, Dickinson and Company, East Rutherford, NJ, United States). Statistical calculations of mean, standard error of the mean (SEM), one-way ANOVA followed by Sidak’s multiple comparison test were performed using GraphPad Prism version 10.2.3 (GraphPad Inc., La Jolla, CA, United States). Differences between groups were considered significant if *P* < 0.05 (****P < 0.0001).

### RNA isolation and transcriptome sequencing

Macrophage total RNA was purified using Trizol (Invitrogen, Carlsbad, CA, United States) and chloroform separation. Integrity and purity of the RNA samples were evaluated using the Agilent RNA 6000 Nano kit on a 2100 Bioanalyzer system (Agilent Technologies, Santa Clara, CA, United States). RNA concentration was estimated by fluorometry using Picogreen (Invitrogen, Carlsbad, CA, United States) and a Victor 3 multilabel plate reader (PerkinElmer, Waltham, MA, United States). The TruSeq RNA v2 sample preparation kit (Illumina, San Diego, CA, United States) was used for generating libraries of complementary DNA (cDNA) fragments from polyadenylated [poly(A)] RNA. A NovaSeq 6000 sequencer (Illumina, San Diego, CA, United States) was used to sequence cDNA libraries, yielding a total of 100 million 150-bp paired-end reads per sample.

### Mapping and transcript abundance estimation

Alignment of the cDNA-derived reads to the mouse reference genome was performed using HISAT2 (version 2.1.0) [25], following the implemented default parameters for paired-end and non-strand-specific RNA-Seq data. The mouse reference genome (mm10, build name GRCm38) was retrieved from the UCSC Genome Browser.^1^ FeatureCounts (version 1.6.3) [26] was used to estimate transcript abundance from read alignments. Those gene features having a single count across all samples or counts equal to zero were classified as suspected non-expressed genes and further removed before subsequent analyses.

### Data quality assessment and differential expression analysis

For visualization of sample-to-sample distances, gene counts were normalized to correct for differences in sequencing depth. The normalized counts were subsequently transformed to logarithms to the base 2 and the variance stabilizing transformation (VST) algorithm was used to stabilize the variance across the mean [27]. The transformed data was used for calculating all pairwise Euclidean distances and the resulting relationships were visualized by using principal component analysis (PCA).

The DESeq2 R package (version 1.38.1) [28] was used for conducting differential expression analysis on raw counts. C57BL/6-derived counts were taken as the reference for determining the relative difference in basal expression. Therefore, DESeq2 results are interpreted as higher or lower expression levels in BALB/c with respect to C57BL/6. Differentially expressed genes were defined as those having a logarithm to the base 2 of fold change (*log*2FC) of at least 0.5 and a Benjamini- Hochberg (BH) multiple-testing adjusted *P*-value of *<* 0.05. The overall results of the differential expression analysis were visualized using volcano and log ratio and mean average (MA) plots.

### Functional enrichment analysis

The ClusterProfile R package (version 3.12.0) [29] was used for identification of signaling and metabolic pathways defined in the Kyoto Encyclopedia of Genes and Genomes (KEGG) database. A cut-off *P*-value of 0.05 was used to identify KEGG pathways overrepresented in the differentially expressed genes. Two independent lists of genes with higher and lower expression in BALB/c with respect to C57BL/6 were separately used as input files. The non-redundant list of genes contained in all identified enriched KEGG pathways except for those describing pathogen- or disease-specific pathways was extracted. Differences in basal gene expression levels between mouse strains in the non-redundant list of genes were visualized as an expression plot and a heatmap based on the RNA-Seq counts normalized by size factor and variance stabilizing transformation (VST).

### Quantitative PCR

For quantitative real time (RT)-PCR, 1 µg of total RNA was first reverse-transcribed using the High-Capacity cDNA reverse transcription kit (Applied Biosystems, Waltham, MA, United States). Subsequently, RT-PCR was performed on a QuantStudio 5 thermocycler (Applied Biosystems, Waltham, MA, United States) using SYBR Green PCR Master Mix (Applied Biosystems, Waltham, MA, United States). Cycling conditions consisted of an initial hold at 95°C (10 min) and 40 cycles of 95°C (15 s), and 60°C (60 s). The housekeeping gene encoding hypoxanthine phosphoribosyltransferase (*hprt*) was used as endogenous control for data normalization. Analyses of relative gene expression of *Tnf*, *Il10*, *Il4ra*, *Mrc1*, *Ccl5*, *Fcgr1*, *iNos*, *Sod3* and *Il4* were performed by the 2^(−∆∆CT)^ method. Briefly, cycle threshold (CT) values of the target genes and *hprt* were obtained, and ∆CT values were calculated by substracting the CT values of *hprt* from CT values of the target genes for each sample. The ∆∆CT was calculated using C57BL/6 as reference and substracting its ∆CT from the ∆CT of the BALB/c sample. The relative gene expression of each target gene was then determined using the formula 2^(−∆∆CT)^. The primer sequences used are listed in Supplementary Table S1. Statistical calculations of mean and SEM for fold differences of all target genes from 4 independent experiments were performed using GraphPad Prism version 10-2.3 (GraphPad Inc., La Jolla, CA, United States).

## Results

### RNA sequencing and data quality assessment

To characterize differences in basal expression between BALB/c and C57BL/6, RNA-Seq and differential expression analysis were conducted in unstimulated peritoneal resident macrophages cultured in triplicate. The proportion of macrophages in peritoneal lavages was determined by the percentage of CD11b and F4/80 double positive cells, which was 33.2 ± 3.1% for BALB/c and 41.7 ± 3.0% for C57BL/6 (Supplementary Figure S1). From the double positive population, the proportions of F4/80^Low^CD11b^Low^ and F4/80^High^CD11b^High^ were respectively, 43.2 ± 0.7% and 47.0 ± 1.1% for BALB/c and 30.4 ± 2.5% and 61.3 ± 2.6% for C57BL/6 (Supplementary Figure S1). Total RNA was purified from cells of both mouse strains at 24 h of culture. Sequencing of poly(A)-enriched RNA generated ∼120 million paired-end reads 150-bp long per sample. Sequence data of this project can be retrieved from the Sequence Read Archive (SRA) through BioProject PRJNA656921, accession numbers SRX10075500, SRX10075501 and SRX10075504 for BALB/c samples, and SRX10075508, SRX10075509 and SRX10075510 for C57BL/6 samples. On average, 98% of reads from each mapped unambiguously to the mouse reference genome.

In order to evaluate if our samples from each mouse strain are reasonably different, a principal component analysis (PCA) based on Euclidean distances was performed. For sample distances estimation, data was previously transformed by means of the variance stabilizing transformation (VST) algorithm [27], to correct for sample distance and variance overestimation resulting from calculating [25] the logarithm of small counts. The PCA analysis explained 99% of the total variance and showed a clustering pattern that clearly separates samples according to mouse strain (Supplementary Figure S2).

### Differential expression analysis and pathway enrichment analysis

**FIGURE 1:**
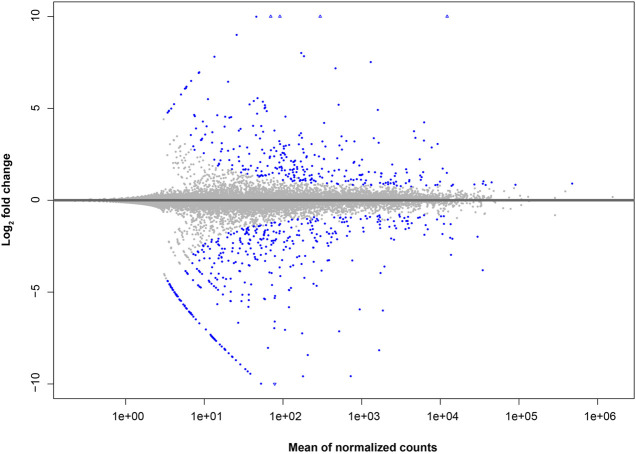
MA-plot of gene expression differences in macrophages of BALB/c in comparison to C57BL/6. The log2 fold change for BALB/c against C57BL/6 samples (*y* axis) is plotted against the average of normalized counts (*x* axis). Each gene is represented with a dot. Genes with significant fold difference between mouse strains (BH multiple testing adjusted *P*-value <0.05) are shown in blue.

For differential expression analysis, the C57BL/6 strain was considered as the reference state. Therefore, our results should be interpreted as higher or lower basal expression levels for genes from BALB/c with respect to C57BL/6, instead of the up- or downregulation of such genes. Altogether, we found 615 differentially expressed (DE) genes between BALB/c and C57BL/6 unstimulated macrophages. From these genes, 266 showed a higher relative basal expression level in BALB/c with respect to C57BL/6 macrophages, whereas 349 showed lower relative basal expression (Figure 1; Supplementary Figure S3; Supplementary Table S2).

**FIGURE 2:**
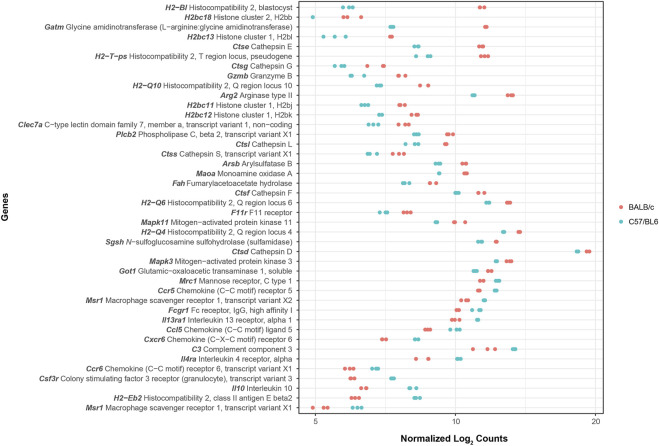
Expression plot of genes in selected enriched KEGG pathways. The plot displays differences in basal gene expression levels between BALB/c (pink) and C57BL/6 (cyan) macrophages for the non-redundant list of genes contained in all identified enriched KEGG pathways except for those describing pathogen- or disease-specific pathways. Data represents log_2_ RNA-Seq counts normalized by size factor and variance stabilizing transformation (VST).

Additionally, we performed a pathway enrichment analysis to identify KEGG pathways enriched by genes with higher and lower basal expression in BALB/c with respect to C57BL/6. In total, 38 KEGG pathways were found to be enriched, 30 and 8 when considering genes with higher and lower basal expression in BALB/c, respectively (Supplementary Table S3). Since we were interested in intrinsic differences between unstimulated macrophages from the two mouse strains, pathogen- or disease-specific pathways (26 in total) were not considered for subsequent analyses. Pathways associated with antigen processing and presentation, cellular senescence and apoptosis, cell adhesion, processing within the lysosome, and metabolism of amino acids were all enriched by genes with higher basal expression in BALB/c with respect to C57BL/6 (Table 1). Conversely, pathways found to be enriched by genes with lower basal expression in BALB/c with respect to C57BL/6 were mainly associated with cytokine-cytokine receptor interaction and hematopoietic cell lineage differentiation (Table 2). In general, pathway enrichment analysis showed that, of all the genes with basal differences in expression between the two mouse strains, 42 appear to be associated with cellular and immune functions that could shed light on macrophage activation patterns (Figure 2; Supplementary Figure S4).

**TABLE 1:** Genes with higher basal expression in BALB/c relative to C57BL/6 macrophages along with their assigned KEGG pathways.

Gene	Product	Fold change
Phagosome/Antigen processing and presentation (mmu04145/mmu04612)^a^		
*H2-Bl*	Histocompatibility 2, blastocyst	185.60
*H2-T-ps*	Histocompatibility 2, T region locus, pseudogene	8.82
*H2-Q10*	Histocompatibility 2, Q region locus 10	5.37
*Clec7a*	C-type lectin domain family 7, member a	3.24
*H2-Q6*	Histocompatibility 2, Q region locus 6	2.41
*H2-Q4*	Histocompatibility 2, Q region locus 4	2.03
Cellular senescence (mmu04218)		
*Mapk11*	Mitogen-activated protein kinase 11/p38 MAPK-β	2.19
*Mapk3*	Mitogen-activated protein kinase 3/ERK-1	1.77
Lysosome (mmu04142)		
*Ctse*	Cathepsin E	10.44
*Ctsg*	Cathepsin G	8.10
*Ctsl*	Cathepsin L	3.12
*Ctss*	Cathepsin S	2.91
*Arsb*	Arylsulfatase B	2.62
*Ctsf*	Cathepsin F	2.46
*Sgsh*	N-sulfoglucosamine sulfohydrolase (sulfamidase)	1.92
*Ctsd*	Cathepsin D	1.90
Neutrophil extracellular trap formation (mmu04613)		
*H2bc18*	H2B clustered histone 18	101.15
*H2bc13*	H2B clustered histone 13	17.54
*Ctsg*	Cathepsin G	8.10
*H2bc11*	H2B clustered histone 11	4.33
*H2bc12*	H2B clustered histone 12	3.60
*Clec7a*	C-type lectin domain family 7, member a	3.24
*Plcb2*	Phospholipase C, beta 2	3.21
*Mapk11*	Mitogen-activated protein kinase 11	2.19
*Mapk3*	Mitogen-activated protein kinase 3	1.77
Cell adhesion molecules (mmu04514)		
*F11r*	F11 receptor	2.30
Arginine and proline metabolism (mmu00330)		
*Gatm*	Glycine amidinotransferase (L-arginine:glycine amidinotransferase)	30.32
*Arg2*	Arginase type II	4.72
*Maoa*	Monoamine oxidase A	2.61
*Got1*	Glutamic-oxaloacetic transaminase 1, soluble	1.76
Apoptosis (mmu04210)		
*Gzmb*	Granzyme B	6.69
Tyrosine metabolism (mmu00350)		
*Fah*	Fumarylacetoacetate hydrolase	2.50

^a^ KEGG pathways database accession numbers are indicated next to the pathway name.

**TABLE 2:** Genes with lower basal expression in BALB/c relative to C57BL/6 macrophages along with their assigned KEGG pathways.

Gene	Product	Fold change
Cytokine-cytokine receptor interaction (mmu04060)^a^		
*Il10*	Interleukin 10	0.16
*Ccr6*	Chemokine (C-C motif) receptor 6	0.28
*Il4ra*	Interleukin 4 receptor, alpha	0.30
*Cxcr6*	Chemokine (C-X-C motif) receptor 6	0.31
*Ccl5*	Chemokine (C-C motif) ligand 5	0.37
*Il13ra1*	Interleukin 13 receptor, alpha 1	0.44
*Ccr5*	Chemokine (C-C motif) receptor 5	0.44
Phagosome (mmu04145)		
*Msr1*	Macrophage scavenger receptor 1	0.05
*H2-Eb2*	Histocompatibility 2, class II antigen E beta2	0.08
*C3*	Complement component 3	0.31
*Fcgr1*	Fc receptor, IgG, high affinity I	0.48
*Mrc1*	Mannose receptor, C type 1	0.53
Hematopoietic cell lineage (mmu04640)		
*Csf3r*	Colony stimulating factor 3 receptor (granulocyte)	0.16
*Fcgr1*	Fc receptor, IgG, high affinity I	0.48

^a^ KEGG pathways database accession numbers are indicated next to the pathway name.

Interestingly, pathways related to the phagosome and mechanisms of antigen processing and presentation were found to be enriched by genes encoding non-classical MHC class I (MHC-Ib) H-2Q molecules, loci 4, 6 and 10 *(H2-Q4*, *H2-Q6* and *H2-Q10*), and their basal expression levels were 2 to 5-fold higher in BALB/c than C57BL/6 (Table 1). Among genes found to have higher basal expression in BALB/c are those encoding cathepsins E, G, L, S, F and D (*Ctse*, *Ctseg*, *Ctsl*, *Ctss*, *Ctsf*, *Ctsd*), arylsulfatase B (*Arsb*) and *N*-sulfoglucosamine sulfohydrolase (*Sgsh*), all of which are associated with lysosomal degradation, ranging from 2 to 10-fold in the case of *Ctse*. Other genes associated with arginine and proline metabolism, including those encoding arginase type II (*Arg2*) and L-arginine:glycine amidinotransferase (*Gatm*) showed basal expression levels of 4.7 and 30 times higher in BALB/c with respect to C57BL/6, respectively. Additional genes with higher expression in BALB/c included those encoding proteins associated with cellular senescence and apoptosis, such as mitogen-activated protein kinases 11/p38 MAPK-β (*Mapk11*) and MAPK3/Extracellular signal-regulated kinase (ERK) 1 (*Mapk3*), with expression levels approximately two times higher, as well as the apoptosis-activating serine protease granzyme B (*Gzmb*), with 6.7-fold expression in BALB/c.

Conversely, genes with lower basal expression in BALB/c were mostly associated with cytokine-cytokine receptor interaction and with the phagosome, showing expression levels that ranged from 0.52 to 0.16 times those of C57BL/6 (Table 2). Genes associated with these pathways include those encoding interleukin 10 (*Il10*), chemokine (C-C motif) ligand 5 (*Ccl5*), chemokine (C-C motif) receptors 5 (*Ccr5*) and 6 (*Ccr6*), chemokine (C-X-C motif) receptor 6 (*Cxcr6*), α subunits of IL-4 and IL-13 receptors (*Il4ra* and *Il13ra1*), mannose receptor C-type 1 (*Mrc1*), and the high-affinity Fc-gamma receptor (*Fcgr1*). Additionally, genes encoding enzymes involved in the regulation of oxidative stress such as glutathione reductase (*Gsr*), superoxide dismutase 3 (SOD3) (*Sod3*), and glutathione-S transferase mu 2 (*Gstm2*) also had a lower basal expression in BALB/c showing respectively expression levels of 0.39, 0.29 and 0.02 times those of C57BL/6 (Supplementary Table S2).

### Relative gene expression and cytometry analyses

**FIGURE 3:**
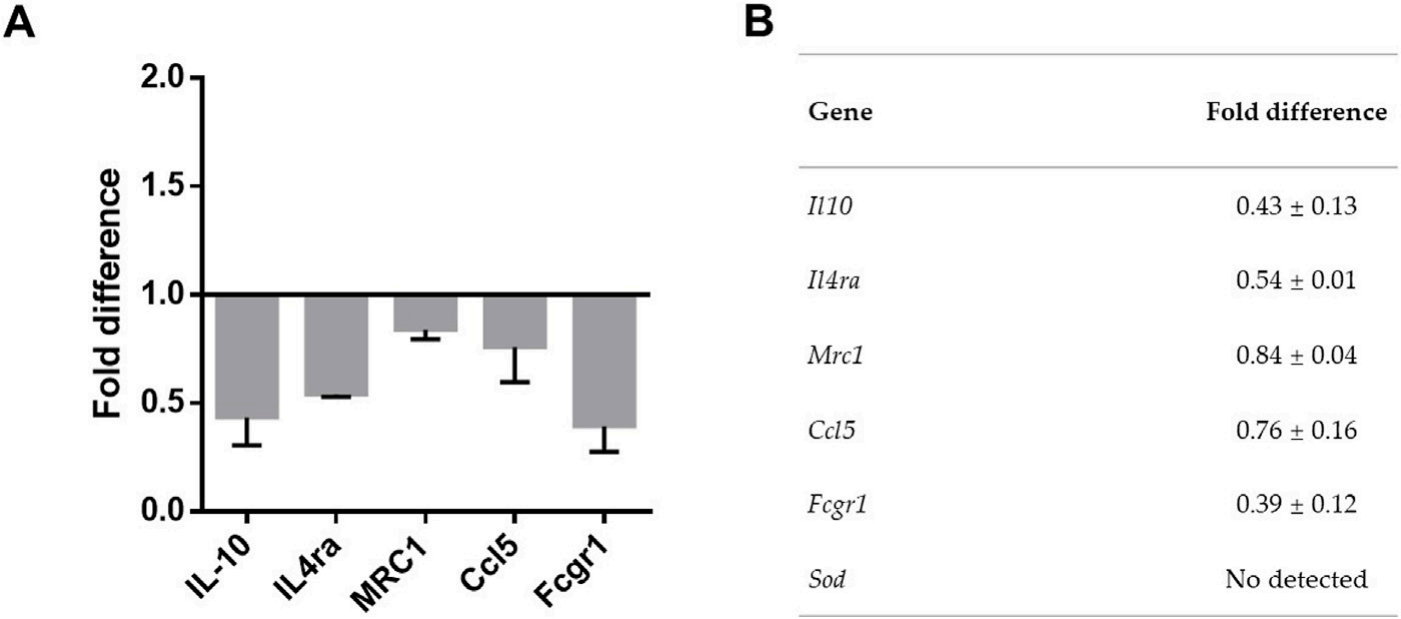
Relative gene expression of selected genes on BALB/c respective to C57BL/6. Adherent cells obtained from peritoneal lavage were cultured for 24 h. Total RNA was obtained and mRNA for *Il10*, *Il4ra*, *Mrc1*, *Ccl5*, *Fcgr1* and *Sod3* determined by quantitative real-time (RT)- PCR. **(A)** Graph shows results normalized to *hprt* expression and are presented as the fold difference of mRNA expression on BALB/c relative to C57BL/6. **(B)** Table shows the results represented by mean ± SEM. Results were obtained from four independent experiments performed in duplicates.

To confirm the transcriptomic findings discussed above, we used RT-PCR to compare the relative expression of a subset of genes on BALB/c cells respective to C57BL/6. We examined genes *Il10*, *Il4ra*, *Mrc1*, *Ccl5*, *Fcgr1* and *Sod3*, all of which exhibited differential expression in the transcriptomic analysis. Consistent with the results obtained in differential expression analysis, cells from BALB/c mice showed a lower expression of *Il10*, *Il4ra*, *Mrc1* and *Fcgr1* in the non-activated condition, when compared to cells from C57BL/6 mice (Figures 3A, B). Meanwhile, *Sod3* was not detected in the examined samples (Figure 3B).

Additionally, we also used RT-PCR to evaluate the relative expression of genes *Tnf*, *iNos*, and *Il4*, which are relevant to the M1 or M2 macrophage phenotypes, although they were not found to be differentially expressed in the transcriptomic analysis. Interestingly, we observed slight differences in the relative expression of these genes between BALB/c and C57BL/6 cells. *Tnf* showed increased expression in BALB/c compared to C57BL/6, while *iNos* and *Il4* exhibited a slight decreased in BALB/c (Supplementary Figure S5). These discrepancies between results from RNA-Seq and RT-PCR are likely associated with inherent features of each methodology and differences in their detection limits [30]. These results suggested that there is not an evident predisposition for the M1 or M2 phenotypes in the cells of these mouse strains.

**FIGURE 4:**
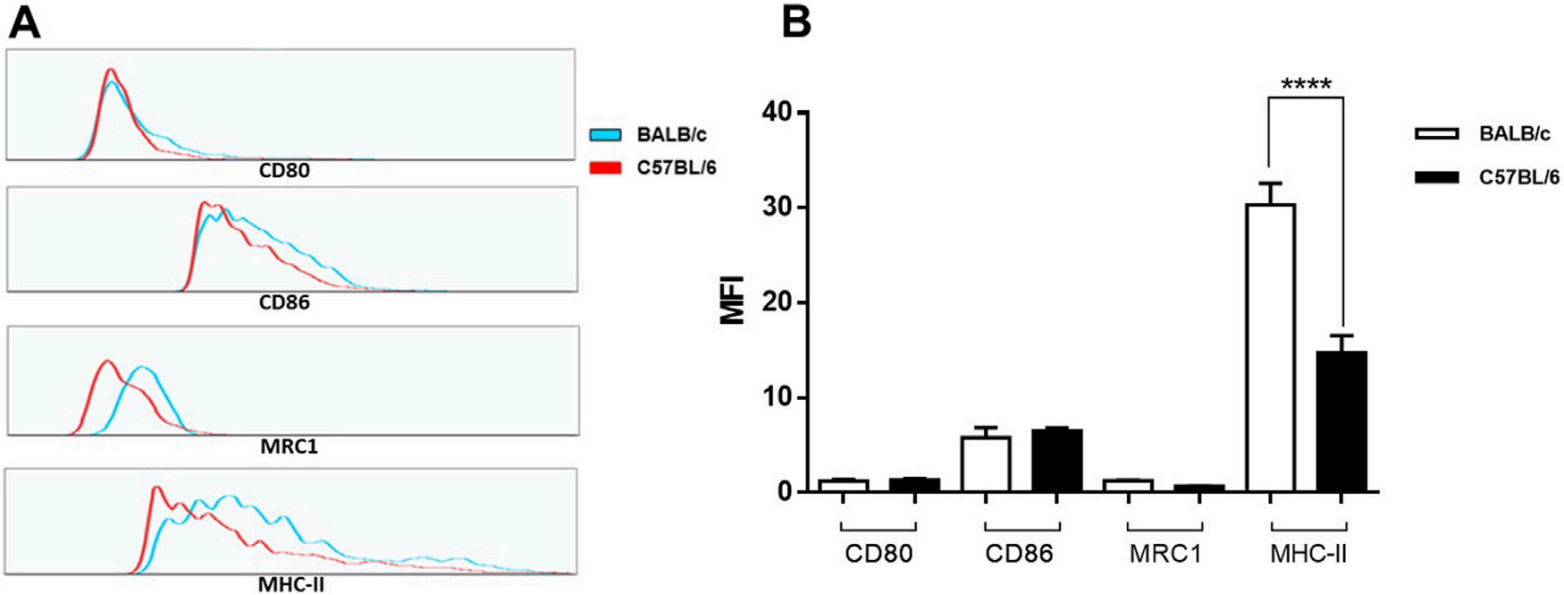
Cell surface marker expression of CD80, CD86, MRC1 and MHC II in unstimulated peritoneal cells from BALB/c and C57BL/6 mice. Adherent cells obtained from peritoneal lavage were culture for 24 h. Surface expression of CD11b, CD80, CD86, MRC1 and MHC-II was analyzed by flow cytometry. Representative histograms **(A)** and bar plots for the mean fluorescence intensity (MFI) **(B)** of each marker on CD11b^+^ positive cells. Bar plots represent mean ± SEM from peritoneal lavages seeded in duplicates from two independent experiments. ****, *p* < 0.0001.

Moreover, to investigate if there was a marker expression pattern indicative of a predisposition towards any specific macrophage phenotypes, we examined the cell surfaces markers expression of CD80, CD86, MRC1 and MHC II in unstimulated peritoneal cells from BALB/c and C57BL/6 mice. CD80 and CD86 are essential for antigen presentation and activation of T cells and are considered M1 phenotype markers [31–33]. Meanwhile, MRC1 is a classical M2 cellular marker [34]. In the CD11b population we did not observe significant differences in the mean fluorescence intensity (MFI) for CD80 or CD86 markers (Figure 4; Supplementary Table S4). These genes were not identified as differentially expressed by the RNA-Seq analysis between cells from BALB/c and C57BL/6 mice. No significant differences in the expression levels of MRC1 were also found between CD11b^+^ BALB/c and C57BL/6 peritoneal cells (Figure 4; Supplementary Table S4). Despite the lower gene expression levels of MRC1 in BALB/c cells compared to C57BL/6, we were unable to detect these differences at the protein level. In the case of MHC II, recognized as a marker for both phenotypes [34] BALB/c cells exhibited significant higher expression than C57BL/6 cells (Figure 4B).

## Discussion

C57BL/6 and BALB/c mice are known to mount opposite T-cell polarization responses that influence a different phenotype of macrophage programs [35]. *In vivo*, C57BL/6 displays a predominant Th1 response to intracellular pathogens, whereas BALB/c displays a Th2 response [36–38]. It has been suggested that BALB/c mice have specific allelic variants of genes encoding immune-related molecules that promote Th2 responses [39, 40]. Available data indicates that macrophages derived from C57BL/6 or BALB/c mice exhibit distinct activation pathways in response to the same stimuli, ultimately steering a characteristic response of the M1 and M2 phenotypes, respectively [2, 8, 9]. We have studied herein the baseline gene expression patterns of resident peritoneal macrophages from BALB/c and C57BL/6 mice to provide insights into whether the cells of these mouse strains might exhibit a predisposition to any of the macrophage phenotypes. Ultimately, we are interested in understanding if this baseline gene expression could affect the interpretation of results concerning differential gene expression analysis in further presence of pathogens colonizing macrophages.

Our results showed a lower basal expression of M2-related genes, such as IL-4Rα, in BALB/c macrophages. IL-4Rα can bind IL-4 and IL-13, promoting the expression of M2-associated genes. In the absence of this receptor both IL-4- and IL-13-mediated functions are compromised [41]. *In vivo*, IL-4Rα deficiency in macrophages has been shown to reduce the number of M2 macrophages in the liver in a pathogen-free mouse model of induced fibrosis [42]. An IL-4Rα-independent mechanism of Th2 differentiation has also been observed in IL-4Rα-deficient BALB/c mice after infection with *L. major* [43]. Meanwhile, *in vitro*, Th2 differentiation appears to be affected by the absence of IL-4Rα [43]. In macrophages, the IL-4 produced by these cells acts in an autocrine manner through the IL-4Rα receptor; however, IL-4 does not appear to be relevant for the *in vitro* differentiation of the M2 phenotype under type II activation [44]. We have previously observed an upregulation of the gene encoding IL-4 in BALB/c resident peritoneal macrophages in response to infection with *L. panamensis*, but no changes were found in IL-4Rα expression levels [23]. Since the expression of IL-4Rα is known to be very low in macrophages [44], the lower basal expression of this receptor found here in resting BALB/c cells is not likely to determine the phenotype of macrophages in response to *in vitro* intracellular pathogens.

We also observed a lower basal expression for the M2-related marker, the type 1 mannose receptor C (MRC1), in unstimulated BALB/c cells relative to C57BL/6 cells. MRC1 appears to be an essential regulator of glycoprotein homeostasis [45]. It has been observed that the lack of MRC1 is related to the upregulation of pro-inflammatory cytokines in endotoxemia models [46]. Additionally, MRC1 expressed on M2 macrophages has been implicated as a mediator of the infection with virulent strains of some pathogens, such as *Mycobacterium tuberculosis* and *Leishmania*, increasing the severity of the disease [47, 48]. The lower basal expression of this and other M2-related genes observed here for BALB/c, associated with a higher basal expression in C57BL/6, is consistent with a previous study where increased M2-related marker expression was reported for C57BL/6 resident peritoneal macrophages in the absence of specific stimulation [49]. It has been suggested that tissue microenvironment favors MRC1 expression in tissue resident macrophages in both, mice and humans [50, 51]. At the protein level, however, no differences in the surface expression of MRC1 was observed between peritoneal cells of BALB/c and C57BL/6 mice. These findings are consistent with other studies demonstrating that, in unstimulated human PBMC-derived macrophages, the expression levels of MRC1 do not follow the pattern observed in the MRC1 marker gene expression [33]. These results collectively suggest that these mouse strains do not show a predisposition for either macrophage phenotype.

Although a higher basal expression of genes encoding cathepsins and other lysosomal enzymes was observed here in BALB/c cells relative to C57BL/6, previous data showed downregulation or no changes in the expression of these genes in response to *L. panamensis* infection [23]. Cathepsins are involved in antigen processing and thus influence immune responses [52, 53]. Cathepsin L, which was found to be increased at the basal level in BALB/c cells, seems to be involved in the generation of a Th1 response against *Leishmania* [54]. We also observed a higher basal expression of cathepsin D in BALB/c cells. The role of cathepsin D in the modulation of immune response is not well understood; however, it has been shown that the inhibition of cathepsin D during *L. major* infection affects both Th1 and Th2 responses [55]. Thus, BALB/c macrophages appear to be genetically prepared to generate a response favoring the resolution of *Leishmania* infection, nevertheless, these cells tend to show an inability to efficiently respond to infection during host-parasite interaction. Indeed, BALB/c cells expressed similar levels of cell surface markers characteristic of the M1 phenotype, such as CD80 and CD86, to those expressed by C57BL/6 cells. Moreover, surface expression levels of MHC II were higher in BALB/c than in C57BL/6 CD11b^+^ peritoneal cells. Since overexpression of MHC II is found in both M1 and M2 phenotypes [34], these results support the idea that BALB/c is well-equipped to respond to inflammatory stimuli. However, there seems to be no evident predisposition for any of the macrophage phenotypes in the cells of both mouse strains in their basal state.

Interestingly, BALB/c showed a higher basal expression of genes encoding non-classical MHC class I (MHC-Ib) molecules H2-Q4 (Qb-1), H2-Q6 and H2-Q10. Although, no specific role has been defined for H2-Q4 (Qb-1) and H2-Q6 [56], H2-Q10 has been recently identified as a ligand of the inhibitory Ly49C receptor, suggesting that it may act as a regulator of NK cell activation [57]. Additionally, H2-Q10 has been found to have high affinity for CD8αα, thus possibly playing a role in the control of liver-resident CD8αα γδT cells [58]. Moreover, H2-Q10 stabilizes the expression of the MHC-1b molecule H2-T23 (Qa-1b), the murine homolog of HLA-E. This fact suggests that H2-Q10 may have additional immune-modulating effects. Specifically, antigen presentation through Qa-1b has been shown to induce an upregulation of the inhibitory NK receptors CD94/NKG2A in secondary infections with *Listeria monocytogenes*. This leads to reduced apoptosis of cytotoxic T-lymphocytes and a prolonged cytotoxic response [59]. As recent data has demonstrated that H2-Q10 has multiple roles that are relevant to the regulation of immunity, further research is required to determine if the higher basal expression of H2Q molecules in BALB/c mice macrophages may contribute to the response to infections with intracellular pathogens.

Among the genes with a lower basal expression in BALB/c, which indicates a higher expression in C57BL/6 is the high-affinity Fc-gamma receptor, which is important for taking opsonized pathogens. It has been previously reported that thioglycolate-elicited macrophages from C57BL/6 mice also express higher basal levels of this receptor [60]. Other genes found to have a higher basal expression level in C57BL/6 were those encoding pro-inflammatory chemokines and chemokines receptors. Although the gene encoding the IL-10 anti-inflammatory cytokine was also found to have a higher basal expression, which was confirmed by RT-PCR. Despite the higher expression of both anti- and pro-inflammatory genes, it has been previously shown that macrophages from C57BL/6 mice infected with *Leishmania* exhibit a gene expression pattern consistent with an M1 phenotype and have lower levels of intracellular amastigotes than BALB/c cells [23]. These findings suggest that C57BL/6 macrophages are intrinsically prepared to battle infections with intracellular pathogens such as *Leishmania*.

Although not found to be associated with any specific KEGG pathway, genes encoding enzymes involved in antioxidant programs such as superoxide dismutase 3 (SOD3), glutathione reductase and glutathione-S transferases also showed high expression in C57BL/6 compared to BALB/c. SOD3 is an important antioxidant enzyme, which prevents the formation of peroxynitrite (ONOO^−^) through superoxide (O_2_^−^) -mediated inactivation of NO [61, 62]. Glutathione reductase, on the other hand, preserves the pool of reduced glutathione, which significantly contributes to regulating reactive oxygen species within the cells [63]. Intracellular glutathione levels in antigen-presenting cells influence the pattern of Th1/Th2 cytokines [64]. It has been shown that the induction of glutathione reductase in response to *L. major* infection in C57BL/6 bone marrow-derived macrophages is several times greater than in BALB/c cells [65]. Higher gene expression and protein levels of antioxidant systems in C57BL/6 compared to BALB/c have also been observed at the basal level in bone marrow-derived macrophages [66]. Since higher levels of NO are produced by cells from C57BL/6 animals compared to BALB/c in response to *L. panamensis* infection [23], the results presented herein support the idea that C57BL/6 cells might be better prepared to counteract the negative effects of oxidative stress associated with infection than BALB/c cells.

Since we were working with tissue-resident cell populations, it is important to highlight the role of the microenvironment in the pattern of gene expression and the function of macrophages. Tissue-resident macrophages have a specific genetic signature and can be distinguished by specific gene expression patterns. The tissue microenvironment is essential for establishing macrophage identity [1, 67]. Macrophages populate tissues very early in the embryonic process and it has been suggested that tissue-resident macrophages originate from precursors present in the yolk sac [68–71]. Despite their common origin, these macrophages develop independently, acquiring specialized functions according to the local microenvironment [72–74]. It has been shown that macrophage populations from different organs express unique mRNA transcripts that allow them to perform specialized local functions [1]. However, evidence have shown that mouse resident peritoneal macrophages change their gene expression pattern when transferred to the lungs, assimilating the pattern of the local macrophage population [67]. These data indicate that the tissue microenvironment has the potential to reprogram differentiated macrophages.

It has been shown that *ex vivo* culture of tissue resident macrophages influences the gene expression pattern and activation status of these cells [67, 75, 76]. The transition of macrophages from the peritoneal cavity to culture medium enriched with M-CSF and/or TGF-β results in marked changes in gene expression [75]. Furthermore, the transfer of alveolar macrophages (AM) from the lung microenvironment of ovalbumin-sensitized rats to *ex vivo* culture and their subsequent reintroduction into AM-depleted sensitized rats results in an increase in Th1 cytokines in bronchoalveolar lavage and a reduction in the alveolar hyperresponsiveness [67]. Together these data indicate the importance of the tissue microenvironment in cell activation status and highlight the effect of *ex vivo* culture on cellular responses. Although unstimulated macrophages were culture for 24 h before RNA-Seq analysis in this study, the pattern of gene expression was markedly different from that obtained in response to infection under the same experimental conditions in a previous study [23]. This emphasizes the importance of considering the basal pattern of gene expression for the subsequent analysis of the changes induced in the presence of a stimulus in an *ex vivo* experimental setting.

We also analyzed the proportion of F4/80^high^CD11b^high^ and F4/80^low^CD11b^low^ in peritoneal lavages of C57BL/6 and BALB/c. F4/80^high^CD11b^high^ cells have been identified as large peritoneal macrophages (LPM) and F4/80^low^CD11b^low^ cells as small peritoneal macrophages (SPM) [76]. Evidence shows that both populations of macrophages coexist in the peritoneal cavity and although they show strong similarities in gene expression pattern [75] they are functionally different [76]. Thus, the differences in the basal pattern of gene expression observed herein might not be influenced by differences in the proportion of these two populations between C57BL/6 and BALB/c cells. However, it has been shown that adhesion to culture dishes induces morphological, phenotypic, and functional modifications of LPM and SPM [76, 77]. This reinforces the need to consider the basal patterns of gene expression when performing differential expression analyses in response to stimuli.

We observed that BALB/c peritoneal cells exhibit a higher mean fluorescence intensity of MHC II compared to C57BL/6 cells. Previous studies have indicated that SPM express elevated levels of MHC II [73, 76] and the numbers of this subset of cells increase in response to inflammatory conditions [78]. The differences in the expression of MHC II observed herein could be related to the varying proportions of SPM and LPM present in both mouse strains [76]. Furthermore, it has been reported that the levels of MHC II expression are dependent on the cell cycle [79]. It has been observed in BMDMs that cell cycle regulators could modulate the basal or activated transcription of genes not directly associated with cell cycle progression [80]. Macrophages arrested in the G_1_ phase of the cell cycle exhibit higher basal expression levels of MHC II; however, they do not increase MHC II expression in response to IFN-γ stimulation [79]. We could speculate that the cell populations analyzed from BALB/c and C57BL/6 mice may have been in different phases of the cell cycle, affecting the basal expression of MHC II. Nevertheless, additional subsets of MHC II^+^ cells have been identified in the peritoneal cavity [81], thus these differences might be also associated with specific cell populations. It has also been suggested that the expression level of certain haplotypes of MHC II molecules in macrophages could promote Th1 differentiation. It has been shown that Th1 bias appears to be influenced by the presence of the I-A^b^ allele [82] Interestingly, this allele is expressed by C57BL/6 mice, while BALB/c express the I-A^d^ allele. The latter emphasizes the need of further studies to characterize in depth resident peritoneal cell populations in these mouse strains.

One limitation of this study is that resident peritoneal cells are a heterogeneous population, thus, we cannot rule out the possible contribution of cells other than macrophages in the observed gene expression patterns. However, the higher proportion of cells in our samples were F4/80^+^CD11b^+^, which are macrophages. Moreover, as tissue resident macrophages could harbour diverse populations of macrophages exhibiting a range of activation states [73, 75], this could contribute to a gene expression pattern that mixes markers for different activation phenotypes. Furthermore, several studies use resident peritoneal cells to understand the mechanisms involved in a variety of disease models where macrophages play an essential role. Hence, the results shown here are relevant for the interpretation of gene expression patterns analysis in the resident peritoneal macrophage population under a pathogen challenge. The impact of baseline gene expression of different tissue-macrophages for the understanding of disease pathogenesis requires further studies.

Globally, the gene expression pattern of uninfected C57BL/6 macrophages exhibited significant differences to that of BALB/c macrophages. Our results suggest that macrophages from each mice strain have a specific basal gene expression pattern, which probably influences, but does not appear to determine by itself, the phenotype in response to pathogens. The final phenotype may be influenced also by post-transcriptional modifications, which in turn could be modulated by the pathogens [83]. This study highlights important aspects to consider when studying the mechanisms involved in the development of resident peritoneal macrophage phenotypes in response to intracellular pathogens.

## Data Availability

The datasets presented in this study can be found in online repositories. The names of the repository/repositories and accession number(s) can be found below: https://www.ncbi.nlm.nih.gov/sra/SRX10075500; https://www.ncbi.nlm.nih.gov/sra/SRX10075501; https://www.ncbi.nlm.nih.gov/sra/SRX10075504; https://www.ncbi.nlm.nih.gov/sra/SRX10075508; https://www.ncbi.nlm.nih.gov/sra/SRX10075509; https://www.ncbi.nlm.nih.gov/sra/SRX10075510.

## Footnotes

1
http://genome.ucsc.edu/

## References

[1] Gautier EL Shay T Miller J Greter M Jakubzick C Ivanov S Gene-expression profiles and transcriptional regulatory pathways that underlie the identity and diversity of mouse tissue macrophages. Nat Immunol (2012) 13:1118–28. 10.1038/ni.2419 23023392 PMC3558276

[2] Mills CD Kincaid K Alt JM Heilman MJ Hill AM. M-1/M-2 macrophages and the Th1/Th2 paradigm. The J Immunol (2000) 164:6166–73. 10.4049/jimmunol.164.12.6166 10843666

[3] Mantovani A Sica A Sozzani S Allavena P Vecchi A Locati M. The chemokine system in diverse forms of macrophage activation and polarization. Trends Immunol (2004) 25:677–86. 10.1016/j.it.2004.09.015 15530839

[4] Yunna C Mengru H Lei W Weidong C. Macrophage M1/M2 polarization. Eur J Pharmacol (2020) 877:173090. 10.1016/j.ejphar.2020.173090 32234529

[5] Arango DG Descoteaux A. Macrophage cytokines: involvement in immunity and infectious diseases. Front Immunol 5. 10.3389/fimmu.2014.00491 PMC4188125 25339958

[6] Verreck FAW de Boer T Langenberg DML Hoeve MA Kramer M Vaisberg E Human IL-23-producing type 1 macrophages promote but IL-10-producing type 2 macrophages subvert immunity to (*myco)bacteria* . Proc Natl Acad Sci U S A (2004) 101:4560–5. 10.1073/pnas.0400983101 15070757 PMC384786

[7] Orecchioni M Ghosheh Y Pramod AB Ley K. Macrophage polarization: different gene signatures in M1(LPS+) vs. Classically and M2(LPS–) vs. Alternatively activated macrophages. Front Immunol (2019) 10:1084. 10.3389/fimmu.2019.01084 31178859 PMC6543837

[8] Soudi S Zavaran-Hosseini A Muhammad Hassan Z Soleimani M Jamshidi Adegani F Hashemi SM. Comparative study of the effect of LPS on the function of BALB/c and C57BL/6 peritoneal macrophages. Cell J (2013) 15:45–54. 23700560 PMC3660024

[9] Santos JL Andrade AA Dias AAM Bonjardim CA Reis LFL Teixeira SMR Differential sensitivity of C57BL/6 (M-1) and BALB/c (M-2) macrophages to the stimuli of IFN-*γ*/LPS for the production of NO: correlation with iNOS mRNA and protein expression. J Interferon and Cytokine Res (2006) 26:682–8. 10.1089/jir.2006.26.682 16978073

[10] Reiner SL Locksley RM. The regulation of immunity to *Leishmania major* . Annu Rev Immunol (1995) 13:151–77. 10.1146/annurev.iy.13.040195.001055 7612219

[11] Lipoldová M Demant P. Genetic susceptibility to infectious disease: lessons from mouse models of leishmaniasis. Nat Rev Genet (2006) 7:294–305. 10.1038/nrg1832 16543933

[12] Fornefett J Krause J Klose K Fingas F Hassert R Benga L Comparative analysis of humoral immune responses and pathologies of BALB/c and C57BL/6 wildtype mice experimentally infected with a highly virulent *Rodentibacter pneumotropicus* (*Pasteurella pneumotropica*) strain. BMC Microbiol (2018) 18:45. 10.1186/s12866-018-1186-8 29848308 PMC5977748

[13] Bertolini TB de Souza AI Gembre AF Piñeros AR Prado Rd Q Silva JS Genetic background affects the expansion of macrophage subsets in the lungs of *Mycobacterium tuberculosis*-infected hosts. Immunology (2016) 148:102–13. 10.1111/imm.12591 26840507 PMC4819146

[14] Oswald IP Afroun S Bray D Petit J-F Lemaire G. Low response of BALB/c macrophages to priming and activating signals. J Leukoc Biol (1992) 52:315–22. 10.1002/jlb.52.3.315 1381743

[15] Dileepan KN Page JC Li Y Stechschulte DJ. Direct activation of murine peritoneal macrophages for nitric oxide production and tumor cell killing by interferon-γ. J Interferon and Cytokine Res (1995) 15:387–94. 10.1089/jir.1995.15.387 7648440

[16] Chakkalath HR Titus RG. *Leishmania major*-parasitized macrophages augment Th2-type T cell activation. The J Immunol (1994) 153:4378–87. 10.4049/jimmunol.153.10.4378 7963516

[17] Gregory DJ Sladek R Olivier M Matlashewski G. Comparison of the effects of *Leishmania major* or *Leishmania donovani* infection on macrophage gene expression. Infect Immun (2008) 76:1186–92. 10.1128/iai.01320-07 18086813 PMC2258831

[18] Shadab M Das S Banerjee A Sinha R Asad M Kamran M RNA-seq revealed expression of many novel genes associated with *Leishmania donovani* persistence and clearance in the host macrophage. Front Cel Infect Microbiol (2019) 9:17. 10.3389/fcimb.2019.00017 PMC6370631 30805314

[19] Rodriguez NE Chang HK Wilson ME. Novel program of macrophage gene expression induced by phagocytosis of *Leishmania chagasi* . Infect Immun (2004) 72:2111–22. 10.1128/iai.72.4.2111-2122.2004 15039333 PMC375188

[20] Ehrlich A Castilho TM Goldsmith-Pestana K Chae W-J Bothwell ALM Sparwasser T The immunotherapeutic role of regulatory T cells in *Leishmania* (*Viannia) panamensis* infection. J Immunol (2014) 193:2961–70. 10.4049/jimmunol.1400728 25098291 PMC4170189

[21] Rocha FJS Schleicher U Mattner J Alber G Bogdan C. Cytokines, signaling pathways, and effector molecules required for the control of *Leishmania* (*Viannia) braziliensis* in mice. Infect Immun (2007) 75:3823–32. 10.1128/iai.01335-06 17517868 PMC1951993

[22] Castilho TM Goldsmith-Pestana K Lozano C Valderrama L Saravia NG McMahon-Pratt D. Murine model of chronic *L*. (*Viannia) panamensis* infection: role of IL-13 in disease. Eur J Immunol (2010) 40:2816–29. 10.1002/eji.201040384 20827674 PMC3289133

[23] Restrepo CM Llanes A Herrera L Ellis E Lleonart R Fernández PL. Gene expression patterns associated with *Leishmania panamensis* infection in macrophages from BALB/c and C57BL/6 mice. Plos Negl Trop Dis (2021) 15:e0009225. 10.1371/journal.pntd.0009225 33617537 PMC7932533

[24] Herrera L Llanes A Álvarez J Degracia K Restrepo CM Rivera R Antileishmanial activity of a new chloroquine analog in an animal model of *Leishmania panamensis* infection. Int J Parasitol Drugs Drug Resist (2020) 14:56–61. 10.1016/j.ijpddr.2020.08.002 32950020 PMC7502791

[25] Kim D Langmead B Salzberg SL. HISAT: a fast spliced aligner with low memory requirements. Nat Methods (2015) 12:357–60. 10.1038/nmeth.3317 25751142 PMC4655817

[26] Liao Y Smyth GK Shi W. featureCounts: an efficient general purpose program for assigning sequence reads to genomic features. Bioinformatics (2014) 30:923–30. 10.1093/bioinformatics/btt656 24227677

[27] Anders S Huber W. Differential expression analysis for sequence count data. Genome Biol (2010) 11:R106. 10.1186/gb-2010-11-10-r106 20979621 PMC3218662

[28] Love MI Huber W Anders S. Moderated estimation of fold change and dispersion for RNA-seq data with DESeq2. Genome Biol (2014) 15:550. 10.1186/s13059-014-0550-8 25516281 PMC4302049

[29] Yu G Wang L-G Han Y He Q-Y (2012). clusterProfiler: an R package for comparing biological themes among gene clusters, OMICS, 16: 284–7. 10.1089/omi.2011.0118 22455463 PMC3339379

[30] Rachinger N Fischer S Böhme I Linck-Paulus L Kuphal S Kappelmann-Fenzl M Loss of gene information: discrepancies between RNA sequencing, cDNA microarray, and qRT-PCR. Int J Mol Sci (2021) 22:9349. 10.3390/ijms22179349 34502254 PMC8430810

[31] Zhou Y Yoshida S Kubo Y Yoshimura T Kobayashi Y Nakama T Different distributions of M1 and M2 macrophages in a mouse model of laser-induced choroidal neovascularization. Mol Med Rep (2017) 15:3949–56. 10.3892/mmr.2017.6491 28440413 PMC5436148

[32] Jaguin M Houlbert N Fardel O Lecureur V. Polarization profiles of human M-CSF-generated macrophages and comparison of M1-markers in classically activated macrophages from GM-CSF and M-CSF origin. Cell Immunol (2013) 281:51–61. 10.1016/j.cellimm.2013.01.010 23454681

[33] Hickman E Smyth T Cobos-Uribe C Immormino R Rebuli ME Moran T Expanded characterization of *in vitro* polarized M0, M1, and M2 human monocyte-derived macrophages: bioenergetic and secreted mediator profiles. PLoS One (2023) 18:e0279037. 10.1371/journal.pone.0279037 36862675 PMC9980743

[34] Martinez FO Gordon S. The M1 and M2 paradigm of macrophage activation: time for reassessment. F1000prime Rep (2014) 6:13. 10.12703/p6-13 24669294 PMC3944738

[35] Watanabe H Numata K Ito T Takagi K Matsukawa A. Innate immune response in Th1- and Th2-dominant mouse strains. Shock (2004) 22:460–6. 10.1097/01.shk.0000142249.08135.e9 15489639

[36] Scott P Natovitz P Coffman RL Pearce E Sher A. Immunoregulation of cutaneous leishmaniasis. T cell lines that transfer protective immunity or exacerbation belong to different T helper subsets and respond to distinct parasite antigens. The J Exp Med (1988) 168:1675–84. 10.1084/jem.168.5.1675 2903212 PMC2189120

[37] Heinzel FP Sadick MD Holaday BJ Coffman RL Locksley RM. Reciprocal expression of interferon gamma or interleukin 4 during the resolution or progression of murine leishmaniasis. Evidence for expansion of distinct helper T cell subsets. The J Exp Med (1989) 169:59–72. 10.1084/jem.169.1.59 2521244 PMC2189187

[38] Hsieh CS Macatonia SE O’Garra A Murphy KM. T cell genetic background determines default T helper phenotype development *in vitro* . The J Exp Med (1995) 181:713–21. 10.1084/jem.181.2.713 7836924 PMC2191880

[39] Beebe AM Mauze S Schork NJ Coffman RL. Serial backcross mapping of multiple loci associated with resistance to *Leishmania major* in mice. Immunity (1997) 6:551–7. 10.1016/s1074-7613(00)80343-x 9175833

[40] Knight JM Lee S-H Roberts L Smith CW Weiss ST Kheradmand F CD11a polymorphisms regulate TH2 cell homing and TH2-related disease. J Allergy Clin Immunol (2014) 133:189–97.e8. 10.1016/j.jaci.2013.03.049 23726040 PMC3842370

[41] Classen A Lloberas J Celada A. Macrophage activation: classical vs. Alternative (2009). p. 29–43. 10.1007/978-1-59745-396-7_3 19347309

[42] Weng S-Y Wang X Vijayan S Tang Y Kim YO Padberg K IL-4 receptor alpha signaling through macrophages differentially regulates liver fibrosis progression and reversal. EBioMedicine (2018) 29:92–103. 10.1016/j.ebiom.2018.01.028 29463471 PMC5925448

[43] Mohrs M Holscher C Brombacher F. Interleukin-4 receptor alpha-deficient BALB/c mice show an unimpaired T helper 2 polarization in response to *Leishmania major* infection. Infect Immun (2000) 68:1773–80. 10.1128/iai.68.4.1773-1780.2000 10722563 PMC97347

[44] La Flamme AC Kharkrang M Stone S Mirmoeini S Chuluundorj D Kyle R. Type II-activated murine macrophages produce IL-4. PLoS One (2012) 7:e46989. 10.1371/journal.pone.0046989 23071691 PMC3465319

[45] Lee SJ Evers S Roeder D Parlow AF Risteli J Risteli L Mannose receptor-mediated regulation of serum glycoprotein homeostasis. Science (1979) (2002) 295:1898–901. 10.1126/science.1069540 11884756

[46] Kambara K Ohashi W Tomita K Takashina M Fujisaka S Hayashi R *In vivo* depletion of CD206+ M2 macrophages exaggerates lung injury in endotoxemic mice. The Am J Pathol (2015) 185:162–71. 10.1016/j.ajpath.2014.09.005 25447055

[47] Schlesinger LS. Macrophage phagocytosis of virulent but not attenuated strains of *Mycobacterium tuberculosis* is mediated by mannose receptors in addition to complement receptors. The J Immunol (1993) 150:2920–30. 10.4049/jimmunol.150.7.2920 8454864

[48] Lee SH Charmoy M Romano A Paun A Chaves MM Cope FO Mannose receptor high, M2 dermal macrophages mediate nonhealing *Leishmania major* infection in a Th1 immune environment. J Exp Med (2018) 215:357–75. 10.1084/jem.20171389 29247046 PMC5748861

[49] Breda J Banerjee A Jayachandran R Pieters J Zavolan M. A novel approach to single-cell analysis reveals intrinsic differences in immune marker expression in unstimulated BALB/c and C57BL/6 macrophages. FEBS Lett (2022) 596:2630–43. 10.1002/1873-3468.14478 36001069

[50] Dupasquier M Stoitzner P Wan H Cerqueira D van Oudenaren A Voerman JSA The dermal microenvironment induces the expression of the alternative activation marker CD301/mMGL in mononuclear phagocytes, independent of IL-4/IL-13 signaling. J Leukoc Biol (2006) 80:838–49. 10.1189/jlb.1005564 16849611

[51] Bellón T Martínez V Lucendo B del Peso G Castro MJ Aroeira LS Alternative activation of macrophages in human peritoneum: implications for peritoneal fibrosis. Nephrol Dial Transplant (2011) 26:2995–3005. 10.1093/ndt/gfq771 21324976

[52] Nakagawa TY Rudensky AY. The role of lysosomal proteinases in MHC class Il‐mediated antigen processing and presentation. Immunological Rev (1999) 172:121–9. 10.1111/j.1600-065x.1999.tb01361.x 10631942

[53] Cresswell P. Proteases, processing, and thymic selection. Science (1998) 280:394–5. 10.1126/science.280.5362.394 9575085

[54] Zhang T Maekawa Y Sakai T Nakano Y Ishii K Hisaeda H Treatment with cathepsin L inhibitor potentiates Th2-type immune response in *Leishmania major*-infected BALB/c mice. Int Immunol (2001) 13:975–82. 10.1093/intimm/13.8.975 11470767

[55] Zhang T Maekawa Y Yasutomo K Ishikawa H Fawzy Nashed B Dainichi T Pepstatin A-sensitive aspartic proteases in lysosome are involved in degradation of the invariant chain and antigen-processing in antigen presenting cells of mice infected with *Leishmania major* . Biochem Biophysical Res Commun (2000) 276:693–701. 10.1006/bbrc.2000.3538 11027533

[56] Palmer MJ Frelinger JA. Widespread transcription of a Qa region gene in adult mice. The J Exp Med (1987) 166:95–108. 10.1084/jem.166.1.95 2439640 PMC2188633

[57] Sullivan LC Berry R Sosnin N Widjaja JML Deuss FA Balaji GR Recognition of the major histocompatibility complex (MHC) class ib molecule H2-Q10 by the natural killer cell receptor Ly49C. J Biol Chem (2016) 291:18740–52. 10.1074/jbc.m116.737130 27385590 PMC5009249

[58] Goodall KJ Nguyen A Matsumoto A McMullen JR Eckle SB Bertolino P Multiple receptors converge on H2-Q10 to regulate NK and γδT-cell development. Immunol Cel Biol (2019) 97:326–39. 10.1111/imcb.12222 30537346

[59] Gunturi A Berg RE Forman J. Preferential survival of CD8 T and NK cells expressing high levels of CD94. The J Immunol (2003) 170:1737–45. 10.4049/jimmunol.170.4.1737 12574337

[60] Probst CM Silva RA B Menezes JP Almeida TF Gomes IN Dallabona AC A comparison of two distinct murine macrophage gene expression profiles in response to *Leishmania amazonensis* infection. BMC Microbiol (2012) 12:22. 10.1186/1471-2180-12-22 22321871 PMC3313874

[61] Sah SK Agrahari G Kim T-Y. Insights into superoxide dismutase 3 in regulating biological and functional properties of mesenchymal stem cells. Cell Biosci (2020) 10:22. 10.1186/s13578-020-00386-3 32128111 PMC7045732

[62] Fukai T Ushio-Fukai M. Superoxide dismutases: role in redox signaling, vascular function, and diseases. Antioxid and Redox Signaling (2011) 15:1583–606. 10.1089/ars.2011.3999 PMC3151424 21473702

[63] Couto N Wood J Barber J. The role of glutathione reductase and related enzymes on cellular redox homoeostasis network. Free Radic Biol Med (2016) 95:27–42. 10.1016/j.freeradbiomed.2016.02.028 26923386

[64] Peterson JD Herzenberg LA Vasquez K Waltenbaugh C. Glutathione levels in antigen-presenting cells modulate Th1 versus Th2 response patterns. Proc Natl Acad Sci U S A (1998) 95:3071–6. 10.1073/pnas.95.6.3071 9501217 PMC19696

[65] Bichiou H Rabhi S Ben Hamda C Bouabid C Belghith M Piquemal D *Leishmania* parasites differently regulate antioxidant genes in macrophages derived from resistant and susceptible mice. Front Cel Infect Microbiol (2021) 11:748738. 10.3389/fcimb.2021.748738 PMC8554229 34722338

[66] Depke M Breitbach K Dinh Hoang Dang K Brinkmann L Salazar MG Dhople VM Bone marrow-derived macrophages from BALB/c and C57BL/6 mice fundamentally differ in their respiratory chain complex proteins, lysosomal enzymes and components of antioxidant stress systems. J Proteomics (2014) 103:72–86. 10.1016/j.jprot.2014.03.027 24704164

[67] Lavin Y Winter D Blecher-Gonen R David E Keren-Shaul H Merad M Tissue-resident macrophage enhancer landscapes are shaped by the local microenvironment. Cell (2014) 159:1312–26. 10.1016/j.cell.2014.11.018 25480296 PMC4437213

[68] Ginhoux F Greter M Leboeuf M Nandi S See P Gokhan S Fate mapping analysis reveals that adult microglia derive from primitive macrophages. Science (2010) 330:841–5. 10.1126/science.1194637 20966214 PMC3719181

[69] Schulz C Perdiguero EG Chorro L Szabo-Rogers H Cagnard N Kierdorf K A lineage of myeloid cells independent of Myb and hematopoietic stem cells. Science (2012) 336:86–90. 10.1126/science.1219179 22442384

[70] Yona S Kim K-W Wolf Y Mildner A Varol D Breker M Fate mapping reveals origins and dynamics of monocytes and tissue macrophages under homeostasis. Immunity (2013) 38:1073–9. 10.1016/j.immuni.2013.05.008 PMC3908543 23273845

[71] Takahashi K. Development and differentiation of macrophages and related cells: historical review and current concepts. J Clin Exp Hematopathology (2001) 41:1–31. 10.3960/jslrt.41.1

[72] Kohyama M Ise W Edelson BT Wilker PR Hildner K Mejia C Role for Spi-C in the development of red pulp macrophages and splenic iron homeostasis. Nature (2009) 457:318–21. 10.1038/nature07472 19037245 PMC2756102

[73] Okabe Y Medzhitov R. Tissue-specific signals control reversible program of localization and functional polarization of macrophages. Cell (2014) 157:832–44. 10.1016/j.cell.2014.04.016 24792964 PMC4137874

[74] Butovsky O Jedrychowski MP Moore CS Cialic R Lanser AJ Gabriely G Identification of a unique TGF-β-dependent molecular and functional signature in microglia. Nat Neurosci (2014) 17:131–43. 10.1038/nn.3599 24316888 PMC4066672

[75] Gosselin D Link VM Romanoski CE Fonseca GJ Eichenfield DZ Spann NJ Environment drives selection and function of enhancers controlling tissue-specific macrophage identities. Cell (2014) 159:1327–40. 10.1016/j.cell.2014.11.023 25480297 PMC4364385

[76] Ghosn EEB Cassado AA Govoni GR Fukuhara T Yang Y Monack DM Two physically, functionally, and developmentally distinct peritoneal macrophage subsets. Proc Natl Acad Sci U S A (2010) 107:2568–73. 10.1073/pnas.0915000107 20133793 PMC2823920

[77] Cassado A de Albuquerque JAT Sardinha LR Buzzo Cd L Faustino L Nascimento R Cellular renewal and improvement of local cell effector activity in peritoneal cavity in response to infectious stimuli. PLoS One (2011) 6:e22141. 10.1371/journal.pone.0022141 21799778 PMC3142143

[78] Kim K-W Williams JW Wang Y-T Ivanov S Gilfillan S Colonna M MHC II+ resident peritoneal and pleural macrophages rely on IRF4 for development from circulating monocytes. J Exp Med (2016) 213:1951–9. 10.1084/jem.20160486 27551152 PMC5030807

[79] Xaus J Comalada M Barrachina M Herrero C Goñalons E Soler C The expression of MHC class II genes in macrophages is cell cycle dependent. The J Immunol (2000) 165:6364–71. 10.4049/jimmunol.165.11.6364 11086074

[80] Sánchez I Dynlacht BD. Transcriptional control of the cell cycle. Curr Opin Cel Biol (1996) 8:318–24. 10.1016/s0955-0674(96)80004-4 8743881

[81] Sohn M Na HY Ryu SH Choi W In H Shin HS Two distinct subsets are identified from the peritoneal myeloid mononuclear cells expressing both CD11c and CD115. Immune Netw (2019) 19:e15. 10.4110/in.2019.19.e15 31281712 PMC6597442

[82] Baumgart M Moos V Schuhbauer D Müller B. Differential expression of major histocompatibility complex class II genes on murine macrophages associated with T cell cytokine profile and protective/suppressive effects. Proc Natl Acad Sci (1998) 95:6936–40. 10.1073/pnas.95.12.6936 9618517 PMC22692

[83] Wilkins-Rodríguez AA Escalona-Montaño AR Aguirre-García M Becker I Gutiérrez-Kobeh L. Regulation of the expression of nitric oxide synthase by *Leishmania mexicana* amastigotes in murine dendritic cells. Exp Parasitol (2010) 126:426–34. 10.1016/j.exppara.2010.07.014 20659463

