# Supplementary material for: Baseline gene expression in BALB/c and C57BL/6 peritoneal macrophages influences but does not dictate their functional phenotypes
Source: Exp Biol Med (Maywood). 2025 Jan 3;249:10377. doi: 10.3389/ebm.2024.10377 (PMC11740880; doi:10.3389/ebm.2024.10377)
Supplement: Supplementary file 2 [file DataSheet1.docx]

**Supplementary data**

**Title:** Baseline gene expression in BALB/c and C57BL/6 peritoneal macrophages influences but does not dictate their functional phenotypes

**Authors:** Carlos M. Restrepo, Alejandro Llanes, Lizzi Herrera, Esteban Ellis, Iliana Quintero and Patricia L. Fernández*

**Figure S1. Proportion of macrophages in peritoneal lavages.** Adherent cells obtained from peritoneal lavage were cultured for 24 hours. Subsequently, cells were stained with anti-F4/80 and anti-CD11b antibodies and analyzed by flow cytometry. Representative dot plots are shown for two independent pools of cells from BALB/c and C57BL/6 mice. Table shows the percentage of F4/80^Low^CD11b^Low^ and F4/80^High^CD11b^High^ cells from BALB/c and C57BL/6 double positive population. Data represent mean ± SEM from peritoneal lavages seeded in triplicates from two independent pools.


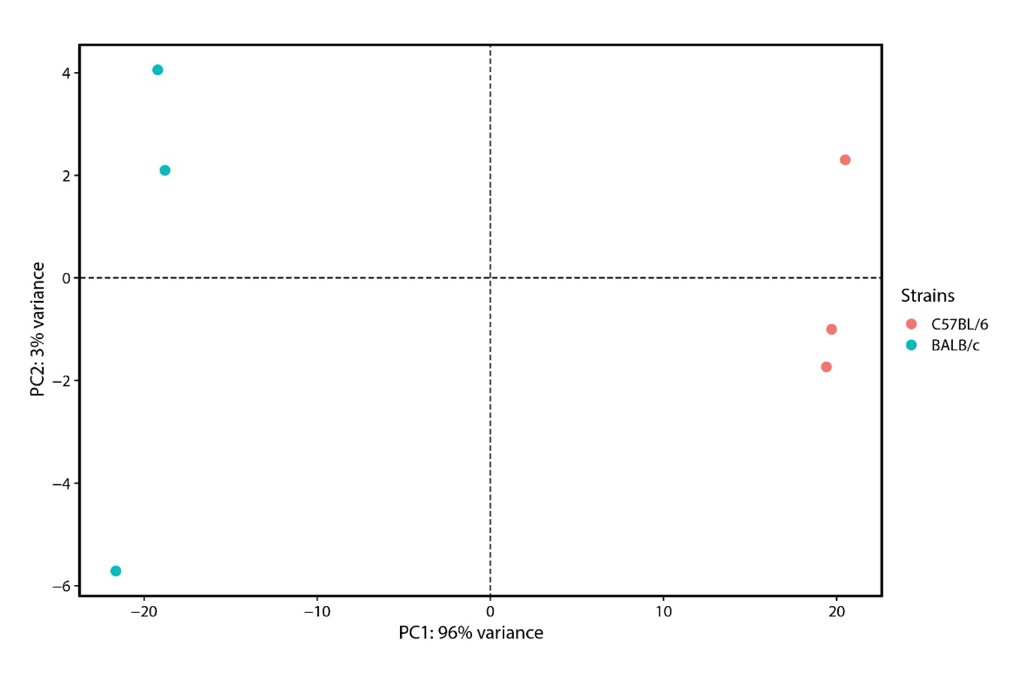


**Figure S2. Two-dimensional PCA plot based on Euclidean distances of VST transformed counts.** The diagram helps to visualize clustering patterns in our samples. The first (PC1; *x* axis) and second (PC2; *y* axis) principal components explain 99% of the total variance and show that samples properly cluster together according to mouse strain (BALB/c or C57BL/6).


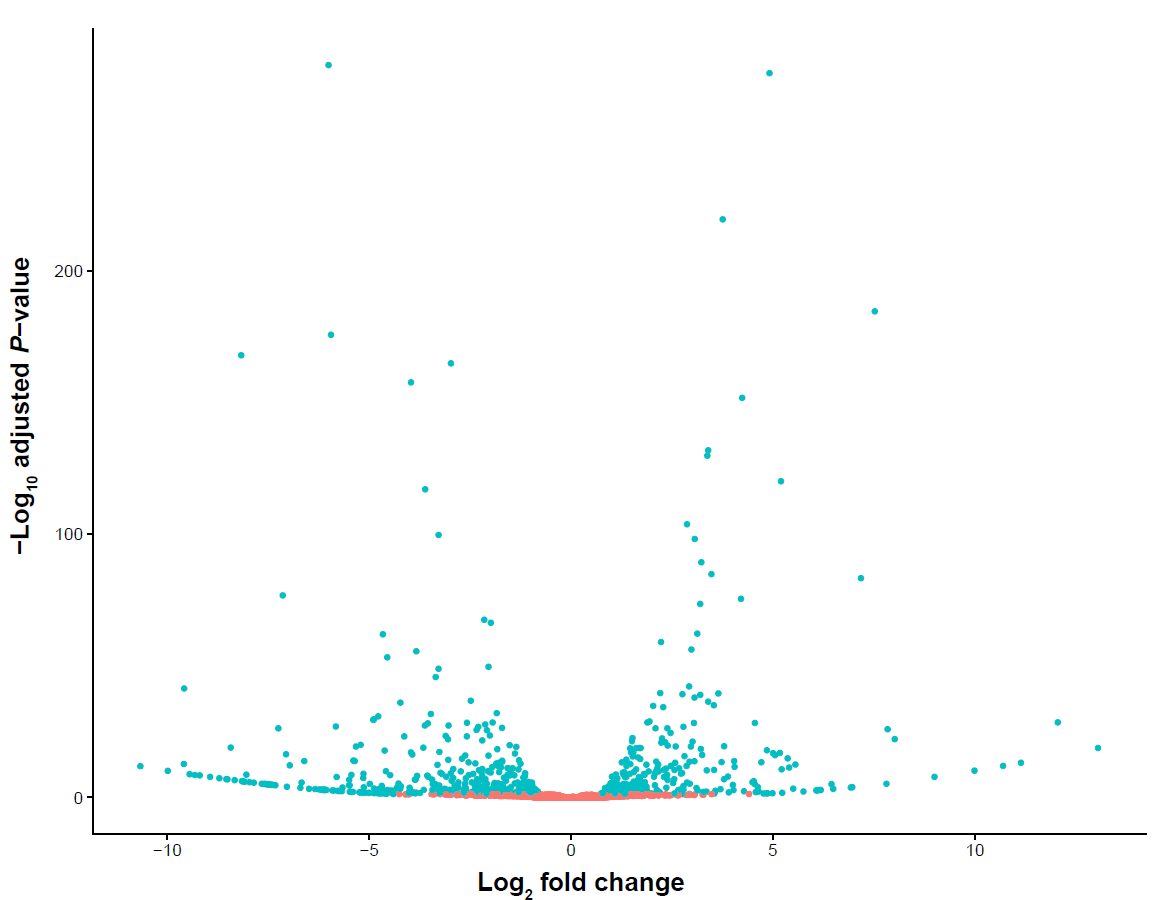


**Figure S3. Differential gene expression analysis between BALB/c and C57BL/6 macrophages.** Volcano plot showing the relationship between fold change and statistical significance for gene expression differences between BALB/c and C57BL/6 macrophages. Each point represents a gene, with log2 fold change on the x-axis and -log10 adjusted *p*-value on the y-axis. Genes with significant fold difference between BALB/c and C57BL/6 macrophages are highlighted in cyan (BH multiple testing adjusted *P* value < 0.05, |log2 fold change| > 0.5). Non-significant genes are shown in pink.


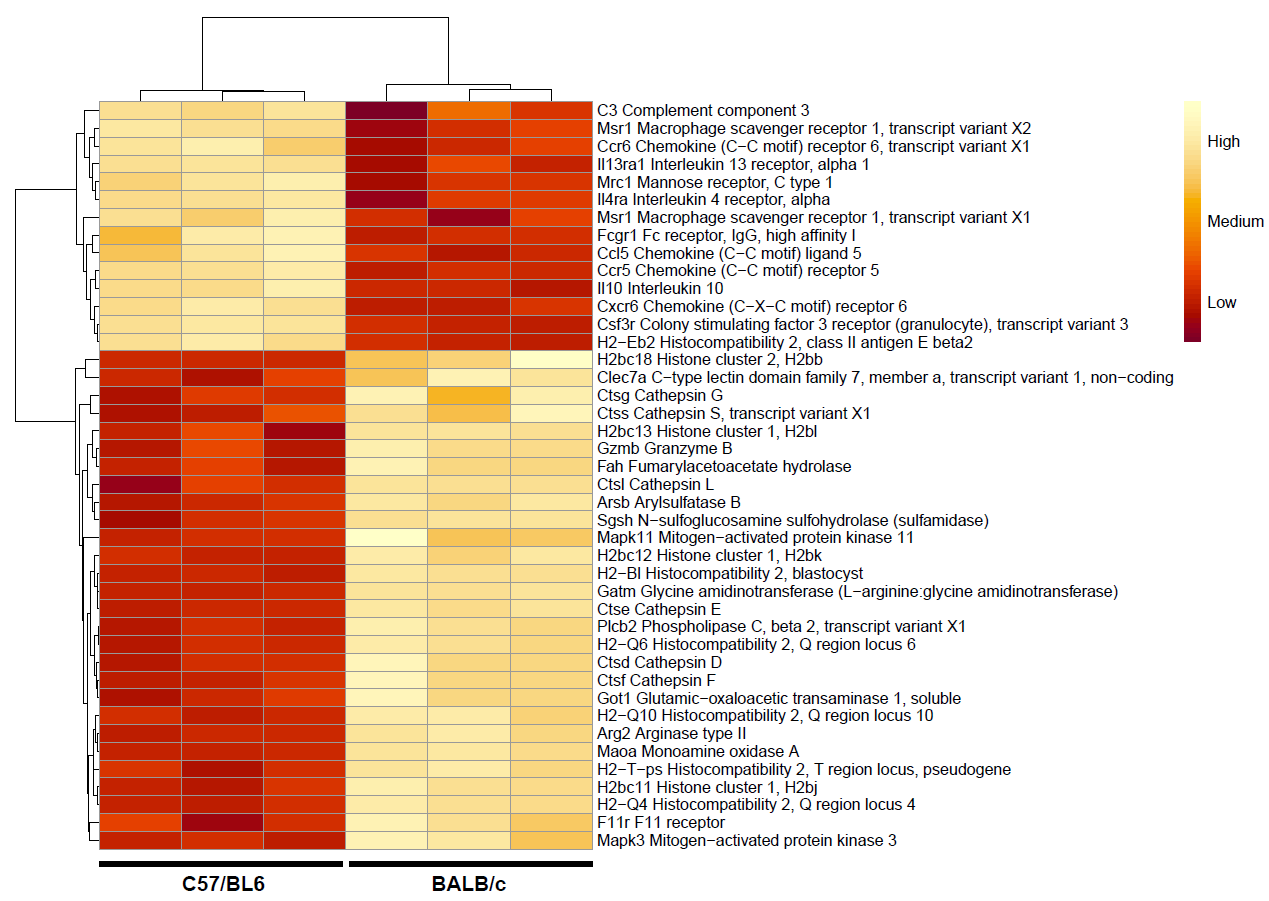


**Figure S4.** **Clustered heatmap of standardized variance stabilizing transformed (VST) RNA-Seq counts of genes in enriched KEGG pathways**. Z-score normalization was applied on transformed counts from the non-redundant list of genes contained in all identified enriched KEGG pathways except for those describing pathogen- or disease-specific pathways. The color scale represents Z-score normalized counts ranging from high (yellow) to low (red) expression. Clustering patterns were determined based on the Euclidean distances of normalized RNA-Seq counts.

**Figure S5.** **RT-PCR relative gene expression on BALB/c cells respective to C57BL/6 of *Tnf*, *iNos* and *Il4*.** Adherent cells obtained from peritoneal lavage were cultured for 24 hours. Levels of mRNA for *Tnf*, *iNos*, and *Il4* were determined by quantitative real-time (RT)- PCR. Results were normalized to *hprt* expression and presented as the fold difference of mRNA expression on BALB/c relative to C57BL/6. Results represent mean ± SEM from peritoneal lavages seeded in duplicates from four independent experiments.

**Table S1.** Primer sequences used in RT-PCR experiments.

| **Gene** | **Forward primer (5’---3’)** | **Reverse primer (5’---3’)** |
| --- | --- | --- |
| *Hprt* | GCTGGTGAAAAGGACCTCT | CACAGGACTAGAACACCTGC |
| *Tnf* | GGTCCCCAAAGGGATGAGAAGTTC | CCACTTGGTGGTTTACTACGACG |
| *Il10* | TAAGGGTTACTTGGGTTGCCAAG | CAAATGCTCCTTGATTTCTGGGC |
| *iNOS* | CCTCCACCCTACCAAGT | CAGCTCCAAGGAAGAGTGA |
| *Il4ra* | TGACCTCACAGGAACCCAGGC | GAACAGGCAAAACAACGGGAT |
| *Fcgr1* | TCCACACAATGGTTTATCAACGG | CACTGTCCTGAAAACTGGCCT |
| *Mrc1* | TGATTACGAGCAGTGGAAGC | GTTCACCGTAAGCCCAATTT |
| *Sod3* | GGCAACTCAGAGGCTCTT C | GTAGCAAGCCGTAGAACAAGA |
| *Ccl5* | AGATCTCTGCAGCTGCCCTCA | GGAGCACTTGCTGCTGGTGTAG |
| *IL-4* | ACAGGAGAAGGGACGCCA | GAAGCCCTACAGACGAGCTCA |

**Table S3.** Full list of KEGG pathways enriched by genes with differential basal expression between BALB/c and C57BL/6 macrophages*.*

| Accession number | KEGG Pathway | No. of DE genes | Pathway size | Adjusted *P* value |
| --- | --- | --- | --- | --- |
| Enriched by genes with higher basal expression in BALB/c | | | | |
| mmu04145 | Phagosome | 13 | 182 | 1.75E-06 |
| mmu05330 | Allograft rejection | 8 | 63 | 5.42E-06 |
| mmu05332 | Graft-versus-host disease | 8 | 63 | 5.42E-06 |
| mmu04612 | Antigen processing and presentation | 9 | 90 | 5.42E-06 |
| mmu05203 | Viral carcinogenesis | 13 | 229 | 5.42E-06 |
| mmu04940 | Type I diabetes mellitus | 8 | 70 | 7.24E-06 |
| mmu05320 | Autoimmune thyroid disease | 8 | 79 | 1.61E-05 |
| mmu05163 | Human cytomegalovirus infection | 12 | 256 | 8.26E-05 |
| mmu05167 | Kaposi sarcoma-associated herpesvirus infection | 11 | 224 | 1.26E-04 |
| mmu04218 | Cellular senescence | 10 | 184 | 1.29E-04 |
| mmu05416 | Viral myocarditis | 7 | 88 | 2.78E-04 |
| mmu05165 | Human papillomavirus infection | 13 | 362 | 3.76E-04 |
| mmu04142 | Lysosome | 8 | 135 | 4.87E-04 |
| mmu05170 | Human immunodeficiency virus 1 infection | 10 | 240 | 9.25E-04 |
| mmu04613 | Neutrophil extracellular trap formation | 9 | 208 | 1.55E-03 |
| mmu04514 | Cell adhesion molecules | 8 | 182 | 3.23E-03 |
| mmu05166 | Human T-cell leukemia virus 1 infection | 9 | 250 | 5.43E-03 |
| mmu05034 | Alcoholism | 8 | 205 | 6.40E-03 |
| mmu00531 | Glycosaminoglycan degradation | 3 | 21 | 9.89E-03 |
| mmu05169 | Epstein-Barr virus infection | 8 | 231 | 1.26E-02 |
| mmu00330 | Arginine and proline metabolism | 4 | 54 | 1.44E-02 |
| mmu04210 | Apoptosis | 6 | 136 | 1.44E-02 |
| mmu04550 | Signaling pathways regulating pluripotency of stem cells | 6 | 140 | 1.60E-02 |
| mmu04916 | Melanogenesis | 5 | 100 | 1.93E-02 |
| mmu05322 | Systemic lupus erythematosus | 6 | 149 | 2.02E-02 |
| mmu05205 | Proteoglycans in cancer | 7 | 205 | 2.15E-02 |
| mmu04934 | Cushing syndrome | 6 | 162 | 2.83E-02 |
| mmu04071 | Sphingolipid signaling pathway | 5 | 124 | 4.17E-02 |
| mmu00350 | Tyrosine metabolism | 3 | 40 | 4.25E-02 |
| mmu05152 | Tuberculosis | 6 | 180 | 4.26E-02 |
| Enriched by genes with lower basal expression in BALB/c | | | | |
| mmu04060 | Cytokine-cytokine receptor interaction | 11 | 292 | 1.42E-02 |
| mmu05134 | Legionellosis | 5 | 61 | 1.43E-02 |
| mmu04061 | Viral protein interaction with cytokine and cytokine receptor | 6 | 95 | 1.43E-02 |
| mmu05143 | African trypanosomiasis | 4 | 39 | 1.93E-02 |
| mmu05150 | Staphylococcus aureus infection | 6 | 128 | 3.74E-02 |
| mmu05152 | Tuberculosis | 7 | 180 | 3.74E-02 |
| mmu04145 | Phagosome | 7 | 182 | 3.74E-02 |
| mmu04640 | Hematopoietic cell lineage | 5 | 94 | 3.86E-02 |

**Table S4.** Percentage and mean fluorescence intensity (MFI) values of CD11b^+^ unstimulated peritoneal cells from BALB/c and C57BL/6 mice expressing CD80, CD86, MRC1 or MHC II cell surface markers.

| **Surface marker*** | **BALB/c** | | **C57BL/6** | |
| --- | --- | --- | --- | --- |
|  | **Percentage** | **MFI** | **Percentage** | **MFI** |
| CD11b^+^CD80^+^ | 10.1 ± 0.7 | 1.2 ± 0.2 | 10.0 ± 0.8 | 1.3 ± 0.2 |
| CD11^+^CD86^+^ | 44.9 ± 2.7 | 5.8 ± 1.1 | 45.0 ± 2.5 | 6.5 ± 0.3 |
| CD11^+^MRC1^+^ | 29.0 ± 1.2 | 1.2 ± 0.05 | 22.8 ± 1.7 | 0.7 ± 0.02 |
| CD11^+^MHC-II^+^ | 7.6 ± 1.7 | 30.3 ± 2.3 | 10.9 ± 1.3 | 14.8 ± 1.8 |

*Results represent mean ± SEM from peritoneal lavages seeded in duplicates from two independent experiments.
